# Positive mental imagery, emotion regulation and depressive symptoms in individuals with alcohol use disorder

**DOI:** 10.3389/fpsyt.2025.1533801

**Published:** 2025-04-08

**Authors:** M. Rydzewska, J. Zaorska, M. Kopera, P. Kobyliński, E. M. Trucco, P. Wiśniewski, A. Marciniuk, A. Żmigrodzka, A. Jakubczyk

**Affiliations:** ^1^ Department of Psychiatry, Medical University of Warsaw, Warsaw, Poland; ^2^ Laboratory of Interactive Technologies, National Information Processing Institute, Warsaw, Poland; ^3^ Department of Psychology and the Center for Children and Families, Florida International University, Miami, FL, United States; ^4^ Department of Psychiatry, Addiction Center, University of Michigan, Ann Arbor, MI, United States; ^5^ Department of Psychiatry, Bielanski Hospital, Warsaw, Poland

**Keywords:** positive mental imagery, vividness, emotional intensity, emotion regulation, flexibility, alcohol use disorder

## Abstract

**Introductions:**

Emotion regulation, depressive symptoms and mental imagery have both been linked to alcohol use disorder (AUD). However, the association between these factors have not been investigated within a group of individuals with AUD.

**Objectives:**

The primary aim of this study was to investigate associations between emotion regulation, depressive symptoms and positive mental imagery among individuals with AUD and healthy controls (HCs).

**Methods:**

The study sample included 136 individuals with AUD and 80 HCs. Severity of depressive symptoms was assessed with the Beck Depression Inventory (BDI) and emotion dysregulation - with the Difficulties in Emotion Regulation Scale (DERS). The Flexible Emotion Regulation Scale (FlexER) was used to measure flexible emotion regulation and the Prospective Imagery Task (PIT) - to assess positive mental imagery.

**Results:**

Vividness of positive mental imagery was significantly lower in the AUD group compared to HC group, while emotional intensity of positive mental imagery was significantly higher in the AUD group. Higher vividness of positive mental imagery was associated with lower emotional dysregulation in the AUD group and with higher flexibility of emotion regulation among both groups. Emotional intensity of positive mental imagery was positively correlated with flexibility of emotion regulation in the AUD group, but negatively correlated with flexibility of emotion regulation among HCs. In the AUD group, both vividness and emotional intensity of positive mental imagery were significantly associated with lower severity of depressive symptoms.

**Conclusions:**

Enhancing positive mental imagery abilities might be a promising strategy in the treatment of AUD.

## 1 Introduction

Mental imagery is defined as perceptual experiences based on memories, in the absence of external sensory input, generally identified as seeing in the “mind’s eye”, hearing in the “mind’s ear”, etc. (1). Neuroscience points towards similarities in brain structures for mental imagery and actual perception (1, 2). Namely, mental imagery plays a crucial role in information processing, particularly in interpreting, reasoning, and problem solving (1) and increases motivation to engage and complete tasks (3). More specifically, positive prospective mental imagery refers to mental simulation of positive future events, so it is thought to reflect pre-experiencing pleasant events (4).

Mental imagery may refer to the vividness, as well as emotional experiencing of imagined situations. The vividness of mental imagery tells us how well the mental imagery imitates perception. On the other hand, the emotional intensity of mental imagery informs the degree of emotional arousal during image creation (5). These two are considered different constructs with different brain areas being responsible for vividness and emotional intensity of mental imagery. Specifically, studies showed that a network of areas, including early and late visual areas (i.e., right parietal cortex, medial frontal cortex and precuneus), is associated with the experienced vividness of visual imagery (6, 7), while the brain regions associated with emotional processing during emotional mental imagery are the dorsomedial prefrontal cortex (mPFC), anterior cingulate cortex (ACC), amygdala and the insular cortex (8).

Mental imagery is viewed as a significant factor influencing substance use by motivating individuals to pursue goals that lead to the experience of pleasurable effects (9). Yet, only a few prior studies have focused on mental imagery among individuals with alcohol use disorder (AUD). Prior work demonstrates that most patients during treatment for AUD experience imagery associated with craving and that the frequency of craving imagery predicts relapse and treatment dropout (4). For example, in a recent study it was observed that cravings were initially dominated by imagery containing the preparative routines and expected outcomes of substance use (10). Other findings from a group of risky drinkers demonstrate that alcohol-related mental imagery (imagining drinking someone’s favorite alcohol) versus neutral imagery (imagining watching someone’s favorite TV show) was rated as more vivid and sensorially rich (11).

A construct that is closely related to mental imagery is cue reactivity. Both relate to cognitive processes that engage memory and emotional reactions. A key difference between these constructs is that cue reactivity refers to the response to external or internal stimuli that trigger specific responses, whereas mental imagery refers to the creation of images in the mind that are unrelated to the current external stimuli (12).

Among individuals with AUD, mental imagery may be used as an internal cue that can trigger the same response as the actual stimulus (external cue). For instance, mentally imagining a glass of wine may increase the urge to drink again, thereby activating cue reactivity. To summarize, mental imagery is a powerful trigger for cue reactivity in individuals with AUD, and it plays a significant role in alcohol cravings and relapse (13, 14). Importantly, there is substantial evidence that individuals with AUD tend to react significantly stronger to alcohol-related cues vs. neutral cues (15). However, future research is needed to confirm results in the area of mental imagery.

Literature examining emotional thinking about the future in individuals with AUD is limited. When exploring the issue of mental imagery, it is worth mentioning the concept of episodic future thinking (EFT). EFT is defined as the ability to imagine or simulate experiences that might occur in one’s personal future. EFT has been associated with adaptive functions, such as planning, goal-directed behavior, and emotion regulation (16).

Hai and collegues observed that patients with AUD have deficits in future thinking. More specifically, these individuals tend to imagine general, rather than episodic, future events that may occur in a specific time and space (17). It has been suggested that individuals with AUD may use excessive generality in future thinking as an avoidance strategy to replace emotional experiences with abstract thought (17). The overgenerality in thinking about the future in AUD was associated with difficulties in solving problems and imagining achievements, which, through a negative impact on self-esteem and motivation, resulted in relapses (17). In fact, prior work demonstrated lower episodic positive, negative, and neutral thinking about the future in participants with AUD compared to a control group. Participants with AUD also showed lower episodic positive and negative future thinking compared to neutral future thinking (18). Interestingly, in another study, positive and near-future events were described in more detail in individuals with AUD than negative and distant future events (19). Also, among individuals with AUD, Noel et al. (19) observed reduced calibration between subjective and objective measures of EFT (19). It has been suggested that it may reflect difficulties with mental imagery and verbalization of imagined content (19).

Another factor essential for development, course, and treatment of AUD is emotion regulation. Emotion regulation is defined by Gross (20) as the process by which individuals influence what kind of emotions they have, when they have them, and how they are experienced and expressed (20). Gratz and Roemer (21) describe emotion regulation as a multidimensional construct involving understanding and acceptance of emotions, control of impulsive behavior, and the use of appropriate emotion regulatory strategies (21). Emotion dysregulation contributes to the development of AUD, affects its course, increases severity of negative affect, promotes the consequences of alcohol use, and increases the risk of relapse (22, 23). It has been consistently reported that patients with AUD have difficulties with accepting their emotions, coping, and avoiding impulsive behavior (24).

The association between mental imagery and emotions plays a significant role in psychopathology (25). There is growing evidence that recruiting mental imagery induces stronger effects on positive or negative emotions and is perceived more realistically than verbal processing (26, 27). It has been suggested that imagination of possible positive future events may facilitate planning, decision-making, and self-regulatory behavior (28), what is significant for emotion regulation. Individuals with minimal or absent imagery vividness may not find imagery to be an effective tool for emotional regulation (29). Furthermore, prior work supports links between dysfunctional mental imagery and mental disorders, such as anxiety disorders, depression, or addictive behaviors (4).

It is well established that poor emotion regulation is associated with higher severity of depressive symptoms. Consistently, greater depressive symptoms have been associated with a lower ability to generate positive future events and a greater ability to generate negative future events (30–32). Moreover, depressive symptoms were found to be associated with less specific, vivid, and detailed mental images of positive (but not negative) future events (30, 33). It was also shown that people with depressive symptoms generate mental images more slowly and are more likely to use the observer’s perspective than the first-person perspective when describing their mental images, which has been associated with lower emotional impact (30, 33, 34). Reduced imagery vividness for positive future events has also been found to discriminate between participants with dysphoria compared to participants without dysphoria (35). Another study demonstrated that individuals who have a higher inclination to generate positive mental imagery during spontaneous future thinking, rather than negative imagery, are more likely to experience a decrease in negative mood due to their stronger sense of optimism (36).

Positive imagery training has been shown to increase positive affect across healthy individuals (26) and individuals with mood disorders (37), reduce dysphoria among depressed and non-depressed patients (38) and reduce symptoms of depression (39). Results from a meta-analysis indicate that imagining the future has a stronger impact on affect than remembering the past (40).

Research on positive EFT has shown that instructing participants to think about achieving positive future goals reduces alcohol cravings in people with AUD (41). Positive EFT was also shown to reduce alcohol consumption and increase the use of protective behavior strategies among college students who drink heavily (42). A recent study investigated whether personalized online functional imagery training (FIT) that instructs participants to apply positive mental imagery in response to negative affect would improve drinking outcomes in hazardous drinking among students. It was observed that FIT increased self-efficacy of control over negative affect drinking, increased control over alcohol consumption, and decreased social drinking motives (43).

In summary, there is indirect support for a connection between mental imagery, emotion regulation, depressive symptoms and problematic alcohol drinking. Individuals with AUD were shown to have no difficulty with creating mental images related to alcohol. On the other hand, imagining situations and images unrelated to drinking might be impaired in this group. The ability to imagine something in the future makes it easier to pursue it, so creating images of drinking may encourage drinking. However, difficulty in mental imagery unrelated to alcohol may result in impaired emotion regulation, deterioration of mood and, consequently, seeking alcohol as a coping strategy to relieve negative affect.

Nevertheless, research on the association between these factors among individuals with AUD is limited. A better understanding of these associations in an AUD sample is essential, particularly considering the significant influence that emotion regulation, depressive symptoms and mental imagery may exert on the development and course of AUD. The current study investigates associations between emotion regulation, depressive symptoms, and positive mental imagery among individuals with AUD. We believe that examination of these factors will likely have notable clinical significance and may help to refine AUD treatments. We focused on positive mental imagery because in our view, treatment using positive compared to negative mental imagery is more acceptable for patients and for this reason it may be associated with better treatment adherence (44). We hypothesized that among individuals with AUD, positive mental imagery would be significantly less vivid compared to controls. Furthermore, we hypothesized that more vivid positive mental imagery would be associated with less intense depressive symptoms and better emotion regulation among individuals with AUD.

## 2 Materials and methods

### 2.1 Individuals with alcohol use disorder

The current data comes from a study examining the behavioral and emotional functioning of individuals with AUD and a healthy control (HC) sample. The study sample included 136 individuals aged 43.21 ± 11.0 who participated in an inpatient alcohol treatment program. Treatment included an 8-week abstinence-based program with intensive individual and group therapy based on a cognitive-behavioral approach. The facility where the study was conducted is situated in the center of Warsaw, Poland. It is devoted specifically to the treatment of individuals with AUD without comorbid substance use disorder of other psychoactive substances. The AUD group consisted of individuals currently receiving treatment for AUD without acute withdrawal symptoms. Patients enrolled in this specific program do not receive benzodiazepines, or any medications that are typically used to treat AUD (e.g., naltrexone, acamprosate etc.). In terms of drinking characteristics, participants reported: (1) first experience of being “drunk” at the age of 17.0 ± 4.3; (2) first experiencing drinking problems during early adulthood (26.1 ± 9.1 years of age); (3) the duration of last drinking period – 143.3 ± 500.1 consecutive days of drinking; (4) the duration of alcohol abstinence prior to completing the study procedures was 49.2 ± 45.1 days. In this group, 44.4% of patients were undergoing the treatment program for the first time, 26.6% for the second time, and 29.0% for the third or more times. The diagnosis of AUD was initially determined by a psychiatrist using the International Classification of Diseases and Related Health Problems 10th Revision (1992) upon the patient’s admission for treatment. This diagnosis was later confirmed through the MINI International Neuropsychiatric Interview (45). Adults with a history of psychosis, current co-occurring mental health disorders requiring medication, current co-occurring substance use disorder (assessed with MINI International Neuropsychiatric Interview) other than nicotine, or a clinically significant cognitive deficit (< 25 on the Mini-Mental State Examination) (46) were not eligible. Research procedures were performed during the first two weeks following treatment admission. The study questionnaires were all assessed within the same study visit.

### 2.2 Healthy controls

The HCs included 80 adults (average years of age = 43.87 ± 14.33) who consulted a general practitioner for an annual physical examination or to seek medical advice concerning prophylactics of somatic diseases (e.g., diet, vaccinations, etc.). This part of the study was conducted in a general practioner’s office located in the center of Warsaw, Poland. Participants who endorsed harmful alcohol use as assessed via the Alcohol Use Disorders Identification Test (AUDIT; 47) were not eligible for study participation. For the HC group, as for the AUD group, the MINI International Neuropsychiatric Interview was utilized to exclude current problematic use of substances other than alcohol.

The current study adopted ethical principles outlined in the Declaration of Helsinki in 1964. Moreover, the Bioethics Committee of the institution where the study took place approved the study procedures.

### 2.3 Measures

#### 2.3.1 Sociodemographic information

Sociodemographic characteristics (e.g., age, biological sex, education) were queried with a self-report survey.

#### 2.3.2 Alcohol use

A modified version of the Substance Abuse Outcomes Module (48) was used to determine the duration of problematic alcohol use, the number of consecutive days of heavy drinking, and the length of abstinence from alcohol use prior to the assessment among individuals with AUD.

#### 2.3.3 Depressive symptoms

Severity of depressive symptoms was assessed with the Beck Depression Inventory (49). The Cronbach’s α for the total BDI score is 0.95.

#### 2.3.4 Emotion dysregulation

The total score from the Polish version of the Difficulties in Emotion Regulation Scale (DERS) (21, 50) was used to assess emotion dysregulation. Difficulties of Emotion Regulation Scale (DERS) measures six domains: (1) lack of acceptance of one’s own emotions, (2) the extent to which negative emotions interfere with one’s ability to focus on tasks, (3) difficulties in impulse control when experiencing negative emotions, (4) difficulties in recognizing one’s own emotions, (5) limited access to effective strategies for regulating one’s emotions and (6) difficulties in describing one’s emotions. A higher score indicates worse emotion regulation. The Cronbach’s α for the total DERS score is 0.92.

#### 2.3.5 Emotion regulation flexibility

Research on emotion regulation (ER) emphasizes differentiation between adaptive and maladaptive ER strategies. However, recent studies point to significance of ER flexibly, which enables choosing between regulation strategies depending on the situations, their effectiveness and consequences. Flexible emotion regulation was measured using Flexible Emotion Regulation Scale (FlexER) (51). This 26-item questionnaire assesses the participants choice of different emotion regulation strategies and whether the strategy is successful. An example strategy is to think differently in order to reduce negative feelings or not to show feelings to others. (e.g., “I keep my feelings under control by using the right strategy in the right situation.”; “I do know how to successfully regulate my feelings in a particular situation.”; “Even when emotions are very intense, I have different strategies to influence them.”). The answers are provided on a 5-point Likert scale anchored from “perfectly agree “ - “ neutral “ - to “ not agree at all “. The Cronbach’s α value for emotion regulation was 0.96.

#### 2.3.6 Positive mental imagery

The Prospective Imagery Task (PIT) (52) was utilized to assess positive mental imagery. In this task individuals were asked to form a mental image of 10 positive future scenarios (e.g., ‘‘You will do well in your course’’) and rate the image’s vividness (PIT_v_p score) and emotional intensity (PIT_e_p score) on a 5-point Likert scale anchored from: (1) no image at all; (2) vague and dim; (3) unclear but recognizable; (4) moderately vivid; and (5) very vivid. The Cronbach’s α for vividness (0.95) and emotional intensity (0.91) was good.

### 2.4 Data analysis

First, an analysis of variance (ANOVA) was used to compare the two groups (AUD and HCs) across the positive mental imagery task subscales (i.e., emotional intensity and vividness). Additionally, to assess the role of biological sex, a two-way ANOVA for each of the two key dependent variables (i.e., vividness and emotional intensity) with group and biological sex as between-subjects factors was conducted.

Second, correlations between positive mental imagery subscales, emotion regulation, and severity of depressive symptoms were conducted separately for the AUD and HC groups.

In posthoc analysis, Hayes’ (2022) PROCESS SPSS macro for moderation was applied to test whether the pattern of opposing directions of associations (if observed) between emotion regulation and emotional intensity or vividness of imaginations would hold, when controlling for age, biological sex and severity of depressive symptoms. For these analyses non-standardized coefficients are reported. Moderation was considered a *post-hoc* analysis in this study, as no specific hypotheses regarding the differences across groups could be formulated based on the lack of prior work comparing AUD samples to HCs.

## 3 Results

### 3.1 Comparison between groups

The between group comparison revealed that vividness was significantly lower in the AUD group compared to HC group, while emotional intensity was significantly higher in the AUD group. Results of the between group comparison is presented in **Table 1**.

**Table 1:** Comparison between individuals with alcohol use disorder and healthy controls.

	AUD	HC	*p*
Age	43.21±11.0	43.87±14.33	0.71
Biological sex (% men)	81%	35%	<.001
Vividness of positive mental imagery	32.32±9.9	37.64±7.0	<.001
Emotional intensity of positive mental imagery	29.99±11.0	23.73±11.4	<.001
Depressive symptoms severity (BDI)	6.96±7.62	20.70±11.85	<.001
Education	13.85±4.73	12.57±5.26	0.16

AUD, alcohol use disorder; HC, healthy controls; BDI, Beck Depression Inventory.

The two-way ANOVA with vividness as the dependent variable revealed a significant group × biological sex interaction: *F*(3,136) = 4.52, *p* = .005, *η_p_* ² = .091. T-tests indicated that male participants with AUD had significantly lower vividness scores (*M* = 32.63, *SD* = 9.27) compared to male participants in the HC group (*M* = 39.48, *SD* = 6.64), *t*(75) = 3.21, *p* = .002, *d* = .79. Among females, the AUD group (*M* = 32.71, *SD* = 9.08, n = 14) also showed lower vividness than their HC counterparts (*M* = 36.16, SD = 7.19), although this difference did not reach statistical significance, *t*(61) = 1.49, *p* = .14, *d* = 0.45.

For emotional intensity, a similar group × biological sex interaction was observed, *F*(3,136) = 7.60, *p* <.001, *η_p_²* = .144. T-tests indicated that male participants with AUD (*M* = 30.81, *SD* = 9.63) scored higher than male participants in the HC group (*M* = 20.39, *SD* = 9.98), *t*(75) =(4.30), p <.001, *d* = 1.07. Female participants with AUD (*M* = 34.50, *SD* = 8.55) likewise scored higher than their HC counterparts (*M* = 26.84, *SD* = 11.51), *t*(61) = 2.31, *p* = .024, *d* = 0.70, although the magnitude of this difference was smaller than in males.

Taken together, these findings indicate that individuals with AUD show lower vividness and higher emotional intensity of positive mental imagery, but these differences are at least partially moderated by biological sex. In particular, the males evidenced a larger effect size in both outcomes than females.

### 3.2 Analysis of correlation

#### 3.2.1 Associations between positive mental imagery and emotional regulation

Within the AUD group, higher vividness of positive mental imagery was significantly associated with lower emotional dysregulation and higher emotion regulation flexibility. Emotional intensity of positive mental imagery was significantly associated with higher flexibility of emotion regulation. Neither level of education nor any of the drinking characteristics (i.e., onset of drinking problems, number of consecutive drinking days during the last drinking period, duration of abstinence prior to the assessment) in the AUD group were significantly associated with either emotion regulation or aspects of positive mental imagery.

In the HC group, vividness of positive mental imagery was significantly associated with higher flexibility of emotion regulation, while emotional intensity of positive mental imagery was significantly correlated with lower flexibility of emotion regulation.

#### 3.2.2 Associations between positive mental imagery and depressive symptoms

In the AUD group, both vividness and emotional intensity of positive mental imagery were significantly associated with lower severity of depressive symptoms. No significant correlations were observed in the HC group.

Correlations for study variables are presented in **Table 2**.

**Table 2:** Correlation matrix for associations between mental imagery and emotion regulation and depressive symptoms.

		Emotion dysregulation		Flexible emotion regulation		Depressive symptom severity	
		AUD	HC	AUD	HC	AUD	HC
Vividness of positive mental imagery	r	**-.203**	0.155	**.286**	**.783**	**-.248**	.088
	p	**.029**	0.172	**.001**	**<.001**	**.007**	.440
Emotional intensity of mental imagery	r	-.081	-0.086	**.226**	**-.675**	**-.189**	-.045
	p	.388	0.452	**.012**	**<.001**	**.041**	.692

Bolded texts refer to p values lower than 0.05.

### 3.3 Posthoc analysis

We also ran a *post-hoc* moderation analysis to test whether AUD status was a significant moderator of the association between flexibility of emotion regulation and emotional intensity of positive mental imagery to confirm differences across groups. The moderation analysis confirmed that when controlling for age, biological sex, and severity of depressive symptoms, AUD status was a significant moderator of the association between flexibility of emotion regulation and emotional intensity of positive mental imagery. The model explained 31% of the variance in emotional intensity of positive mental imagery (*R^2^* = 0.310; *F* [6, 179] = 13.403; *p* < 0.001). A significant interaction was found between flexibility of emotion regulation and AUD status (*b* = 0.670; 95% CI = [0.457, 0.884]; *p* < 0.001; *ΔR^2^* = 0.148). When probing the interaction, findings indicate that the simple slopes for the regression of emotional intensity of positive mental imagery on flexibility of emotion regulation were statistically significant for both groups. Yet, the slope was negative among the HC group (*b* = −0.528; 95% CI = [−0.695, −0.360]; *p* < 0.001) and positive among the AUD group (*b* = 0.143; 95% CI = [0.016, 0.270]; *p* = 0.028).

## 4 Discussion

The main goal of this study was to investigate the association between emotion regulation and vividness, as well as emotional intensity of positive mental imagery among individuals with AUD compared to HCs. Previous research, yet scarce, consistently points to the important role of mental images in the course of addictive disorders (4, 10, 11). Numerous studies also provide strong evidence indicating that emotion dysregulation is a strong risk factor for the development and severe course of AUD (22–24). Importantly, a few recent studies demonstrate that individuals with AUD have deficits in emotional thinking about the future (17, 18). However, to the best of our knowledge, there have been no studies focusing on the association between the ability to generate mental imagery and emotion regulation in patients with AUD.

As expected, individuals with AUD reported less vivid positive mental imagery compared to HCs in our study. On the other hand, emotional intensity of positive mental imagery was higher among individuals with AUD compared to HCs. These differences were present among both men and women, yet more pronounced in males.

Consistent with our hypothesis, higher vividness of positive mental imagery was associated with better emotion regulation (lower emotional dysregulation and higher flexibility of emotion regulation) among individuals with AUD and with higher flexibility of emotion regulation among controls. Among individuals with AUD, vividness of positive mental imagery was also significantly associated with lower severity of depressive symptoms.

Interestingly, associations between emotional intensity of positive mental imagery differed across groups. Namely, emotional intensity of positive mental imagery was positively correlated with flexibility of emotion regulation among individuals with AUD, but negatively correlated with flexibility of emotion regulation among HCs. Moreover, even when controlling for age, biological sex, and depressive symptom severity, AUD status was a significant moderator of the association between flexibility of emotion regulation and emotional intensity of positive mental imagery. In addition, among individuals with AUD, higher emotional intensity of positive mental imagery was associated with lower severity of depressive symptoms.

Our study adds to the existing literature, which suggests a significant deficit of positive mental imagery among individuals with AUD. Prior work indicates that patients with AUD have deficits in future thinking, which means they tend to imagine general rather than episodic future events (17, 53). Similarly, in our observations, the vividness of positive mental imagery among individuals with AUD was significantly lower compared to HCs.

When examining the emotional intensity of mental imagery, we observed the opposite result. Among individuals with AUD, the emotional intensity of positive mental imagery was significantly higher than in HCs. Previous research by Hai et al. (2019) showed that excessive generality in future thinking among individuals with AUD is a reflection of an avoidance strategy to replace emotional experiences with abstract thought (17). In our study, we did not find support for this view as emotional intensity of positive mental imagery was higher among individuals with AUD group compared to HCs. We observed that although patients with AUD had less vivid positive mental imagery, they did not avoid experiencing emotions, and even reported experiencing them with greater emotional intensity compared to HCs. It is possible that the differences between our results and those reported in prior work are due to the fact that the participants in our study were undergoing intensive therapeutic interventions focused on emotions and their experience (i.e., group cognitive-behavioral therapy and biography writing).

In line with studies conducted in other clinical and non-clinical groups (26, 37), we noticed that higher vividness of positive mental imagery was significantly associated with better emotion regulation (lower emotional dysregulation and higher flexibility of emotion regulation) among individuals with AUD. Our findings are generally in agreement with previous work, but to our knowledge this is the first empirical study demonstrating this association with a sample of individuals with AUD. Positive mental imagery was shown to be associated with better emotion regulation and lower negative affect (26, 30), which potentially represent protective factors in the context of problematic alcohol use (54, 55). Surprisingly, we did not observe any significant association between alcohol use variables (e.g., the onset of drinking problems, the number of consecutive drinking days during the last drinking period, duration of abstinence before the assessment) and emotion regulation or aspects of positive mental images in the AUD group. It is possible that the non-significant association is due to unique AUD group characteristics among participants who were undergoing treatment. For example, more than a half of the AUD group were in treatment for at least the second time. Therapeutic activities taking place during treatment may have resulted in improvements in emotion regulation or mental imaging skills, while actual drinking patterns did not change.

Patients with AUD have a tendency to seek immediate gratification, and (as research shows) one way to reduce this phenomenon is to vividly imagine the future when making decisions using prospective imagery, which involves self-projection to experience future events in advance (56). Imagining specific, especially positive future events, can increase the motivation and effort that individuals put into achieving goals. This in turn leads to replacing impulsive acts with more future-oriented decisions (57). Future-oriented positive mental imagery may effectively help to prepare for exposure by reducing anxiety before and during feared situations (58). It can decrease automatic responses to a stressful situation (59) and enhance positive affect (40). Detailed imagination of constructive behaviors improves problem solving and is associated with higher perceived likelihood of a positive outcome and reduced perceived difficulty in coping with a poor outcome (58). Considering this, the deficit in positive mental imagery observed in individuals with AUD may be an important factor contributing to poor behavioral and emotional control that tends to characterize this clinical population. In this view, positive mental imagery might be an interesting therapeutic target. However, as our study is cross-sectional, the direction of these associations may be reversed. According to the AMAUD model (Autobiographical Memory and Alcohol Use Disorders), emotion regulation disorders may lead to a poor desire to construct detailed memories (i.e., autobiographical over-generality), to update one’s life story with new experiences, roles, and/or self-images (i.e., anterograde amnesia), and/or to generate complex future self-images (60).

As mentioned in the Introduction, among individuals with AUD, mental imagery may be a powerful trigger for cue reactivity, playing a significant role in alcohol cravings and relapse (13, 14). Some studies suggest that alcohol cue reactivity is significantly stronger in males compared to females (61). These findings are consistent with our results showing stronger deficits in positive (non-alcohol) mental imagery in males compared to females. Specifically, it is plausible that in male AUD patients mental imagery may be more vivid and more arousing in reference to alcohol-related cues, but significantly less vivid and less arousing in reference to non-alcohol-related cues, as in our study. Importantly, further studies that employ larger samples with an equal distribution of males and females to formally test biological sex differences are needed to empirically test this observation.

Another observation in our study was that emotional intensity of positive mental imagery was associated with higher flexibility of emotion regulation and lower severity of depressive symptoms among individuals with AUD. Accordingly, emotional intensity of positive mental imagery seems to be especially beneficial among individuals with AUD given its connection with better flexibility of emotion regulation and lower negative affect.

Among HCs, we observed that higher vividness of positive mental imagery was associated with higher flexibility of emotion regulation, similar to individuals with AUD. Yet, nuances emerged when examining the emotional intensity of mental imagery. Among HCs, emotional intensity of positive mental imagery was negatively correlated with flexibility of emotion regulation. Thus, in contrast to individuals with AUD, it is likely that the intensity of positive mental imagery may contribute to difficulties regulating emotions in healthy individuals without AUD.

AUD status significantly moderated the association between flexibility of emotion regulation and emotional intensity of positive mental imagery. We can speculate that high emotional intensity of positive imagery among individuals with AUD may develop as a compensation for the primary deficit of emotion regulation. In this case, emotional imagery, may act to enhance emotion regulation strategies by helping to imagine/experience positive emotions associated with future situations. In healthy people, without primary deficits in emotion regulation, such compensation is not needed. In this case, emotional imagery may perhaps utilize already existing resources needed for successful emotional regulation, which eventually leads to its deterioration. Importantly, in the absence of any studies addressing this issue, these speculations should be treated with caution and hopefully addressed in future studies.

Importantly, in both groups, better vividness of positive mental imagery was associated with better flexibility of emotion regulation. Flexible emotion regulation is defined as the ability to effectively regulate emotions by applying various emotion regulation strategies in different situations depending on the features of a situation and one’s own personality characteristics (62). We may assume that vivid positive imagery allows individuals to visualize a desired outcome and to invoke emotion regulation strategies that are most beneficial in a particular situation and most likely to bring the individual closer to the goal they wish to achieve.

A large body of work indicates that depressive symptoms are associated with less specific, vivid, and detailed positive mental images of future events (30, 33). In our study, the association between lower depressive symptoms and better positive mental imagery was observed among individuals with AUD. Interestingly, this association was not present among HCs. However, in HCs low levels of depression severity were observed, limiting data variability and making it difficult to detect true differences or associations in the data.

Our research indicates a possible need for interventions aimed at increasing the vividness and possibly emotional intensity of positive mental imagery among individuals with AUD. Mental imagery may facilitate emotion regulation by activating internally generated representations that compete with external representations of a stimulus, inhibiting further processing of the external emotion trigger (63). The fact that mental images have a stronger impact on emotions than verbal processing of the same material has been used in the image restructuring technique by presenting alternative positive information in the image modality (i.e., preexisting negative mental images were transformed into positive image rescripting or a new positive image was constructed) (64).

Enhancing positive affect while anticipating positive future events has been considered a viable mechanism for reducing anhedonia (40). Positive Imagery Cognitive Bias Modification (CMB) reduced symptoms of anhedonia and increased positive affect in people with depressive symptoms (33, 65). Imagining specific positive events can increase motivation and encourage individuals to make more thoughtful decisions; thus, it is a beneficial intervention for people who tend to make short-sighted decisions (3, 57). Adaptive mental imagery increases the generation of positive affect, which has been linked to mental resilience (66). Generating images about alcohol and its consumption uses up working memory capacity and this makes it difficult to make a rational decision. In 2012, researchers developed an approach to addiction treatment called Functional Decision Making (FDM), which encourages patients to set visualizable goals that can mentally compete with the desire to use drugs. Instead of using any positive image to disrupt craving images, FDM uses images from the mental imagery process. FDM focuses on images showing the benefits of behavioral control and pleasurable goals that are incompatible with substance use (e.g., a partner’s reactions to returning home sober). When positive effects of control are experienced, projecting memories of actual events into the future is likely to maximize the credibility, vividness, and affective power of these images (67). Recently, the MIRAGE randomized pilot trial attempted to evaluate the usefulness of a Functional Imagery Training (FIT) intervention using mental imagery in people with AUD and liver damage. Unfortunately, most people with alcohol-related liver disease who agreed to participate in this study did not complete it. Findings indicate that better strategies are needed to reinforce participants’ engagement with FIT (68). Nevertheless, enhancing mental imagery abilities might be a promising strategy in the treatment of AUD. Yet, its efficacy has to be studied in future work.

Several limitations of this study should be noted. This is a cross-sectional study that included only participants in an inpatient AUD treatment program. Our sample consisted of patients with severe AUD. Because individuals with AUD do not constitute a homogeneous group, the results cannot be generalized to those with mild or moderate AUD. When collecting data, patients in the study group were at different stages of treatment, so their approach to cravings, symptoms of depression, or other emotional states could have been different depending on how much time they have been enrolled in treatment and their level of involvement in therapy. During the therapeutic process, patients learn that all emotions are important and that there is value in recognizing, analyzing, and talking about emotions. Patients also acquire knowledge that emotions should not be divided into bad and good, even though some may be more or less difficult to experience. Importantly, the 12-step program, the elements of which are infused in treatment within our research center, places value on having patients contribute to helping other people with addictive behaviors (e.g., by participating in research). Therefore, it is possible that participation in the program may improve the ability to recognize and describe one’s own affective states (e.g., mood) or regulate emotions, which potentially could lead to lower DERS scores ​​in the treatment group. Yet, we believe that participants provided honest answers. It is worth emphasizing that the data collection process did not involve staff simultaneously treating patients participating in the study, which eliminated the influence of the patient-therapist relationship on the respondents’ answers and study results. In addition, the patients completed the questionnaires (i.e., BDI, DERS) anonymously; only the PIT questionnaire that assesses mental imagery was administered by members of the research team.

Moreover, our sample constitutes a specific group of people who sought treatment. Research should be expanded to larger community samples that are not in treatment. Furthermore, the sample was primarily male and White. Prior work indicates that women show lower vividness of positive mental images compared to men (69). In our study, given partial moderation effects, we also ran separate t-tests by biological sex. These analyses indicated that group differences for emotional intensity and for vividness were more pronounced among men. Nevertheless, the effect sizes for women were also substantively meaningful. In the light of our observations, further research with more diverse and larger samples sizes, especially females, is needed to formally test biological sex differences. We did not collect information on past history of problematic alcohol use among HCs, therefore we cannot exclude past problematic use of alcohol in this group. There are also limitations with regard to the PIT. More specifically, this task adopts a standardized set of cues and it requires a deliberate generation of answers. Accordingly, the results may not reflect the real world. Second, the PIT is based on a self-report, making it difficult to assess the accuracy of subjective vividness and emotional intensity ratings. Moreover, when conducting the PIT, we did not ask about the likelihood of the images taking place in the future.

### 4.1 Conclusion

Despite these limitations, the current study makes a significant contribution to the literature as it is the first study to investigate the association between positive mental imagery and emotion regulation in individuals with AUD. Our results provide support for a positive direct association between vividness of positive mental imagery and better emotion regulation among individuals with AUD. Furthermore, we observed that emotional intensity of positive mental imagery was correlated with higher flexibility of emotion regulation among individuals with AUD. Moreover, both vividness and emotional intensity of positive mental imagery were associated with lower severity of depressive symptoms among individuals with AUD. Our results support notable differences in positive mental imagery among individuals with AUD compared to HCs. In the absence of previous reports, more studies are needed to confirm these results.

## Data Availability

The raw data supporting the conclusions of this article will be made available by the authors, without undue reservation.
